# Next-Generation GLP-1 Agonist Bioassays: Integrating Precision with Biological Relevance

**DOI:** 10.3390/ijms27188068

**Published:** 2026-09-10

**Authors:** Christine Graf, Julia Reuter, Sabrina Rüggeberg, Sebastian Mohr, Julia Petri, Sylvie Gelinas, Birgit Niederhaus, Ulrich Werner, Nina Strebe, Zeina El Zein, Dirk Usener

**Affiliations:** 1 CMC Bioanalytics, Sanofi R&D, 65926 Frankfurt, Germany; 2 MSAT Analytics, Sanofi M&S, 65926 Frankfurt, Germany; 3 Quality Control, FBC Quality, Sanofi M&S, 65926 Frankfurt, Germany; 4 Bioanalytical Solutions, Cerbaresearch, Laval, QC H7V 4A7, Canada; 5 MSAT Statistics, Sanofi M&S, 65926 Frankfurt, Germany; 6 Diabetes Division, Sanofi R&D, 65926 Frankfurt, Germany

**Keywords:** GLP-1 agonists, mode of action, bioassay, potency, HTRF assay, cell cycle arrest, calcium release, assay-ready cells, oral glucose tolerance test in mice, in vivo cross-verification

## Abstract

Glucagon-Like Peptide-1 (GLP-1) is an incretin family peptide hormone with significant therapeutic applications. This study evaluated multiple functional methodologies for assessing GLP-1 agonist biological activity, encompassing in vitro cell-based bioassays and in vivo systems. We developed and comparatively analyzed cell-based bioassays measuring distinct cellular signaling events, such as β-arrestin recruitment, cyclic adenosine monophosphate (cAMP) elevation, and calcium (Ca^2+^) mobilization. Each assay was systematically evaluated for robustness and quality control applicability. All assay systems demonstrated functionality independent of the cellular mechanism employed. Real-time Ca^2+^ flux measurements confirmed the cell-based assay endpoint data, while cross-verification analyses using the oral glucose tolerance test in mice confirmed the representativeness of the cAMP formats. Initial validation studies revealed inter-assay variations, leading to the selection of the cAMP HTRF assay as the preferred quality control methodology. Further assay refinement and subsequent validation studies confirmed the cAMP HTRF assay’s suitability for quality control applications. This methodology has been successfully implemented in routine release testing of a commercially available GLP-1 agonist.

## 1. Introduction

Glucagon-like peptide-1 (GLP-1) is a peptide hormone that belongs to the incretin family. These hormones regulate insulin secretion after food intake, thereby influencing the glucose concentration in the blood.

GLP-1 (glucagon-like peptide-1) is generated through tissue-specific processing of proglucagon, primarily in neuroendocrine L cells of the gastrointestinal tract (especially the ileum) and in the hypothalamus. Following food intake, increased glucose levels stimulate L cells to release stored GLP-1 into the bloodstream. By binding to GLP-1 receptors, it promotes glucose-dependent insulin secretion from pancreatic β-cells, suppresses glucagon release from α-cells, slows gastric emptying, and reduces hunger and thirst, thereby helping to regulate blood glucose levels. However, active GLP-1 has a very short half-life of less than two minutes because it is rapidly degraded by the enzyme dipeptidyl peptidase-4 (DPP-4) [1,2,3,4,5,6,7].

The idea of using GLP-1 as an antidiabetic agent against type 2 diabetes mellitus was first described in the early 1990s [8,9]. One challenge in using GLP-1 was its short half-life due to rapid degradation by DPP-4. Therefore, one treatment strategy involved the use of DPP-4 inhibitors. In 2006, the drug sitagliptin was first tested in clinical studies, followed by vildagliptin. These DPP-4 inhibitors prevent the degradation of GLP-1, thereby increasing its concentration in the bloodstream [4,6,8,10].

Another approach for managing glucose homeostasis is the use of incretin mimetics. These are incretin-like substances that mimic the action of incretin hormones. They are also referred to as GLP-1 (receptor) agonists because they trigger GLP-1 receptor activation [3,11,12].

The sequences of GLP-1 agonists are either based on human GLP-1 or on exendin-4. Exendin-4 is a polypeptide consisting of 39 amino acids found in the saliva of the North American Gila monster (Heloderma suspectum). Exendin-4 has 53% sequence similarity to native human GLP-1 and was first tested in patients in 2005 [8]. Due to its similarity to human GLP-1, it has high receptor affinity and is largely resistant to degradation by DPP-4 [5,6,8,12,13].

GLP-1 receptor agonists have been primarily used in the treatment of type 2 diabetes and obesity due to their ability to enhance insulin in response to meals and reduce glucagon levels, which lowers blood glucose. They also slow gastric emptying, which helps control blood sugar levels and reduce appetite [3,14]. In addition to their use in type 2 diabetes, GLP-1 agonist substances are used or being investigated for the following:

Weight management: These medications promote weight loss by reducing appetite and slowing gastric emptying, making them effective for obesity treatment [3].

Cardiovascular benefits: Some GLP-1 agonists have been shown to reduce the risk of major cardiovascular events in patients with type 2 diabetes [15].

Pain management: Emerging research suggests potential benefits in treating headache and pain disorders, including migraines and neuropathic pain [14].

Cancer treatment [15,16] and mental health, as these medications act on other bodily systems involved in the pathophysiology of various neurological and psychiatric disorders [17].

The U.S. Food and Drug Administration (FDA) approved the first GLP-1 receptor agonist (exenatide) in 2005. GLP-1 agonist medications currently available on the U.S. market are listed in the table below.


**Active Substance**

**Trade Names**

**Indications**
ExenatideByetta^®^T2D
LiraglutideVictoza^®^/Saxenda^®^T2D and reducing the risk of major adverse cardiovascular events in patients with T2D and established cardiovascular diseaseObesityLixisenatideAdlyxin^®^US, Lyxumia^®^EU *T2D
AlbiglutideTanzeum^®^T2D
DulaglutideTrulicity^®^T2D
Exenatide extended-releaseBydureon^®^T2D
Semaglutide injectionOzempic^®^/ Wegovy^®^T2D and reducing the risk of major adverse cardiovascular events in patients with T2D and established cardiovascular diseaseObesitySemaglutide tabletsRybelsus^®^T2D and reducing the risk of major adverse cardiovascular events in patients with T2D and established cardiovascular diseaseObesityThe table summarizes marketed GLP-1 agonist substances and their indications according to [1,18]. * Lixisenatide has been withdrawn from the markets and is available just as combination product with insulin glargine (Soliqua^®^US/Suliqua^®^EU).

Biological activity builds the basis of the efficacy of a drug and is thereby defined as a critical quality attribute [19,20,21] and should reflect the product’s mechanism of action (MoA) [21,22]. As the potency determination is fundamental for compound efficacy and patient safety, it needs to be accurate and robust for successful development and life cycle management of a therapeutic compound [23].

**Figure 1:**
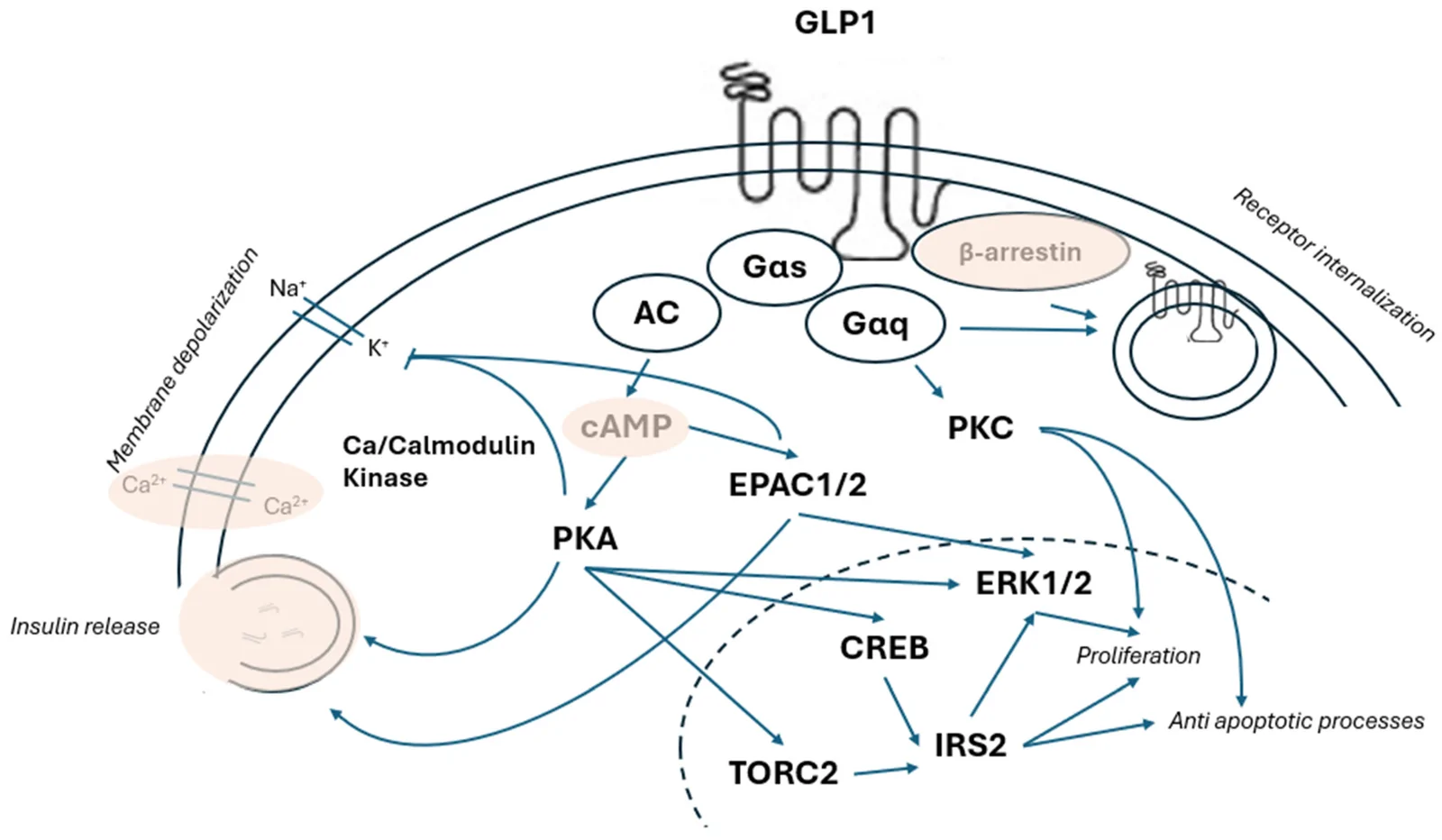
A high-level summary of GLP-1 receptor signaling based on [24,25]. When a GLP-1 agonistic compound binds to the GLP-1 receptor (GLP-1R) on insulin-secreting beta cells, it activates G-protein coupled receptor (GPCR) signaling via Gαs and Gαq. This activation triggers adenylate cyclase (AC) catalysis, resulting in cyclic adenosine monophosphate (cAMP) production. Subsequently, cAMP activates second messenger pathways (PKA and Epac pathways), leading to downstream physiological effects. Alternatively, the receptor activation might result in β-arrestin recruitment. The highlighted parts have been investigated in this study. Insulin secretion is augmented, and plasma glucose levels are decreased in type 2 diabetes mellitus (T2DM).

Figure 1 outlines the mechanism of GLP1 signaling.

GPCRs are the largest and most diverse group of membrane receptors in eukaryotes [26]. These receptors act like an inbox for messages in the form of light energy, peptides, lipids, sugars and proteins. GPCRs, also called seven-transmembrane receptors, interact with G proteins in the plasma membrane. G proteins are specialized proteins with the ability to bind the nucleotides guanosine triphosphate (GTP) and guanosine diphosphate (GDP). They are heterotrimeric, meaning they have three different subunits (α, β, and γ subunits). When a signaling molecule binds to a GPCR, a conformational change in the receptor activates the G protein by replacing GDP with GTP in the α subunit. The G protein dissociates into two parts: the GTP-bound α subunit and the βγ dimer. These two parts are no longer fixed to the receptor but can diffuse laterally in the cell membrane [26,27].

Following G-protein activation, different effector proteins are activated that control the levels of intracellular signaling molecules (e.g., PIP_2_, IP_3_, Ca^2+^, and cAMP). Finally, changes in the levels of these signaling molecules produce alterations in gene transcription or protein activity and result in a functional cellular response [28].

After extracellular binding to the GPCR (GLP-1-R), GDP converts to GTP at the intracellular side of the GPCR. Following Gαs-coupled GPCR activation, active Gαs molecules exert a positive effect on adenylyl cyclase catalysis. cAMP is produced and can bind to protein kinases within the cell, initiating phosphorylation events that regulate target enzymes and transcription factors.

GPCR activation can also be detected through β-arrestin recruitment as an early event in the signaling cascade. This signaling pathway is independent of G-protein activation. The binding of GLP-1 to the receptor on the outside of the cell leads to intracellular phosphorylation of the GPCR. This phosphorylation allows β-arrestin to bind to the receptor inside the cell [29,30].

Another key player involved in the regulation of [cAMP] is the intracellular Ca^2+^ concentration [31]. Cytoplasmic Ca^2+^ release and membrane-potential changes should be investigated using measurement techniques that provide the possibility to obtain real-time data.

In this manuscript, we aimed to compare the sensitivity of different signaling events to GLP-1 activation and subsequently to develop and validate a platform assay for GLP-1 agonists, with special emphasis on representativeness for the mode of action and sufficient precision for use as a QC release test.

## 2. Results

The development of the investigated cell-based bioassays is intended to represent the signaling pathway and the mode of action of the drug to verify the biological efficacy of GLP-1 agonistic compounds. Thus, various cAMP assay systems (ELISA-based, cAMP-Assay of Meso Scale Discovery (MSD), cAMP-Glo™ Assay by Promega or cAMP HTRF^®^ Assay from Cisbio), the β-Arrestin recruitment assay using the PathHunter^®^ platform from DiscoverX and calcium release using the FDSS µcell device from Hamamatsu have been established and tested for robustness, precision and accuracy. The cAMP bioassay in vitro data are compared with in vivo data obtained in an oral glucose tolerance test in female diabetic mouse. Lixisenatide (Adlyxin^®^US, Lyxumia^®^EU) is used in this assay comparison as a representative example for all GLP-1 receptor agonist compounds.

**Figure 2:**
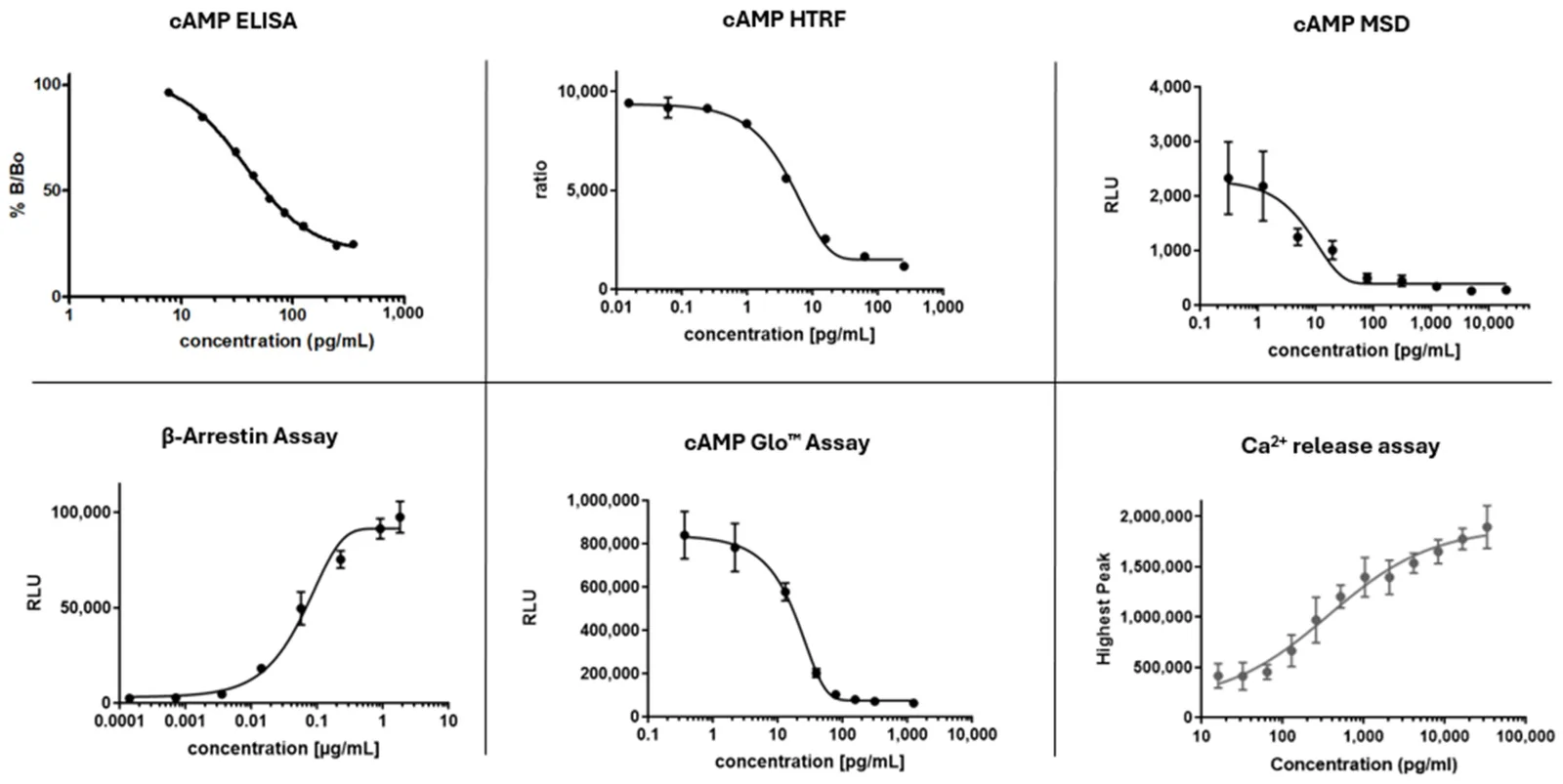
Summary of the dose–response curves of various GLP-1 receptor agonists obtained from cell-based assays in triplicate determinations with standard deviations (8-fold determination for 384-well Ca^2+^ release assay), targeting early- to late-cascade cellular mechanisms and employing different detection methodologies, as indicated above by the respective curves. Increasing lixisenatide concentrations are plotted on the *x*-axis, while the corresponding cellular responses are represented on the *y*-axis.

As shown in Figure 2, all developed assays were functional, regardless of whether early or late events in the GPCR signaling cascade were measured. All evaluated assay platforms demonstrated the capability to generate sigmoidal dose–response curves using lixisenatide as a GLP-1 receptor agonist.

Due to higher variability and an undefined upper asymptote in the measurement data, the cAMP MSD assay was not further evaluated for validation parameters.

Specificity testing against the sample matrix revealed no non-specific signals, confirming that all cell-based bioassays were specific for GLP-1 agonistic activity. For linearity determination, at least five potency levels ranging from 50% to 200% were prepared, and theoretical potency was plotted against measured potency to determine the correlation coefficient (R^2^). Comparison of the linearity results revealed that the PathHunter^®^ β-arrestin assay demonstrated a lower coefficient (R^2^ = 0.87) compared to the other assay systems. The cAMP ELISA followed a slightly different validation strategy, in which the bias and precision of two samples were used to assess linearity.

Accuracy calculations (relative bias of the determined relative potency compared to the theoretical potency level) showed significant variability around the expected value. While the cAMP ELISA, cAMP HTRF, and β-arrestin assays showed the lowest variability around the expected value, the cAMP ELISA showed a slight underestimation—still within an acceptable range. The variations observed were not significant and can be attributed to cellular assay variability. Precision was calculated at 100% potency using six replicate determinations (eight for cAMP HTRF assay) or six determinations at five potency levels for cAMP ELISA. The notably higher values observed in the cAMP ELISA can be explained either by testing at different potency levels or by the implementation of intermediate precision conditions, incorporating different analysts, different cell batches, and different kit lots. All pre-validation data are summarized in Table 1.

### 2.1. Assay Development and Improvement for QC Use

The cAMP HTRF assay was selected for further development among all suitable assay systems due to its low precision variability and its accuracy distribution being closest to zero, despite initially high variability across all methods.

Further optimization efforts were undertaken to enhance robustness and reduce assay variability. Cell growth curves were established to standardize cultivation parameters and support cell banking procedures. Key assay conditions were defined by determining optimal cell density, stimulation duration, and sample dilution across various buffer systems. Additionally, the influence of cell viability and the feasibility of using assay-ready cells was systematically evaluated.

**Figure 3:**
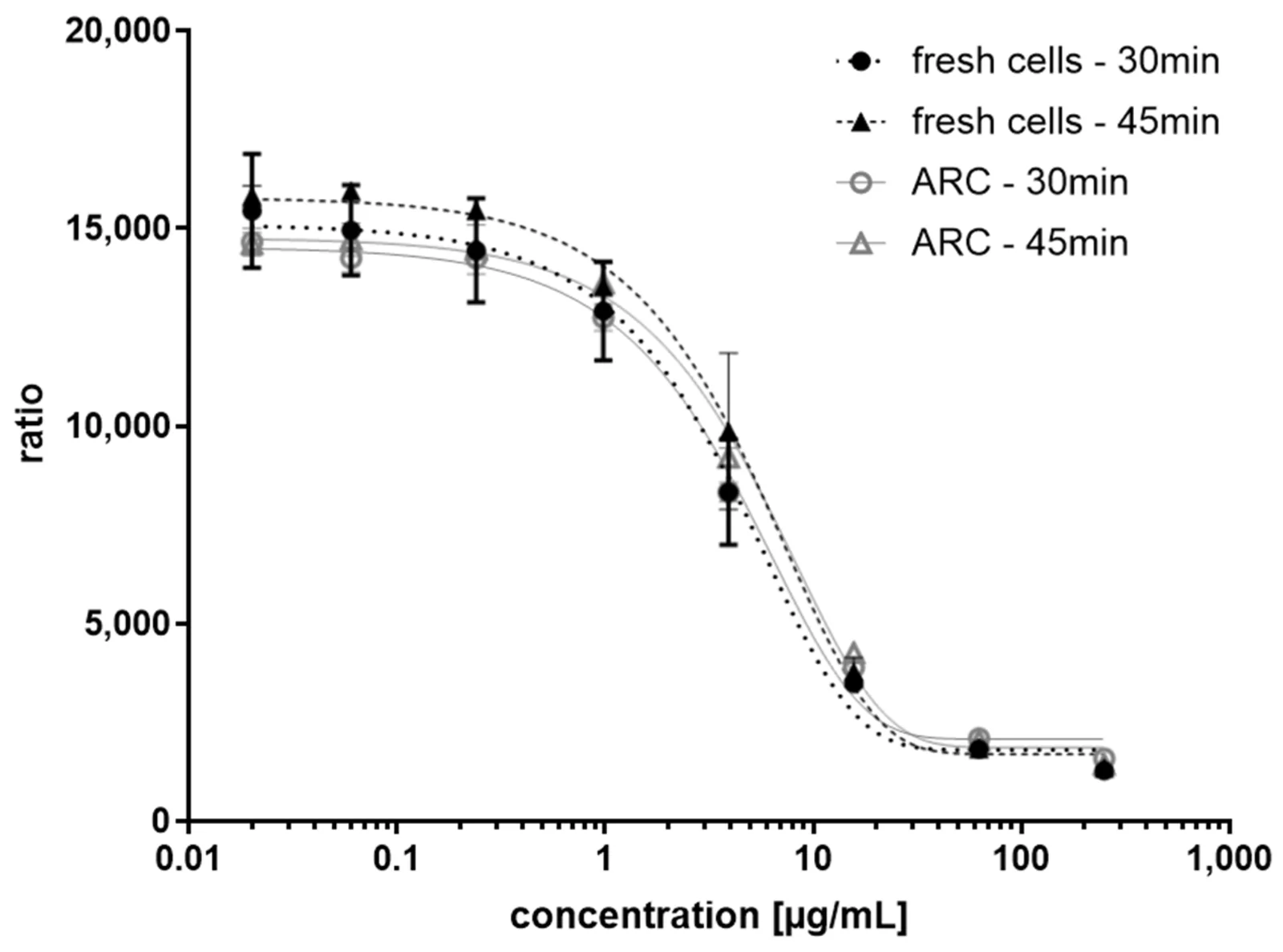
Summary of the dose–response curves using assay-ready cells (shown in gray with solid lines) and cultivated cells (shown in black with dotted lines) in triplicate with SDs. Additionally, different lixisenatide incubation times of 30 min (circles) and 45 min (triangles) were compared, as indicated in the figure.

As shown in Figure 3, the dose–response curves and IC_50_ values are highly comparable using assay-ready cells and cultured cells. Additionally, different incubation times of 30 and 45 min were compared, revealing no significant differences in the final results.

**Figure 4:**
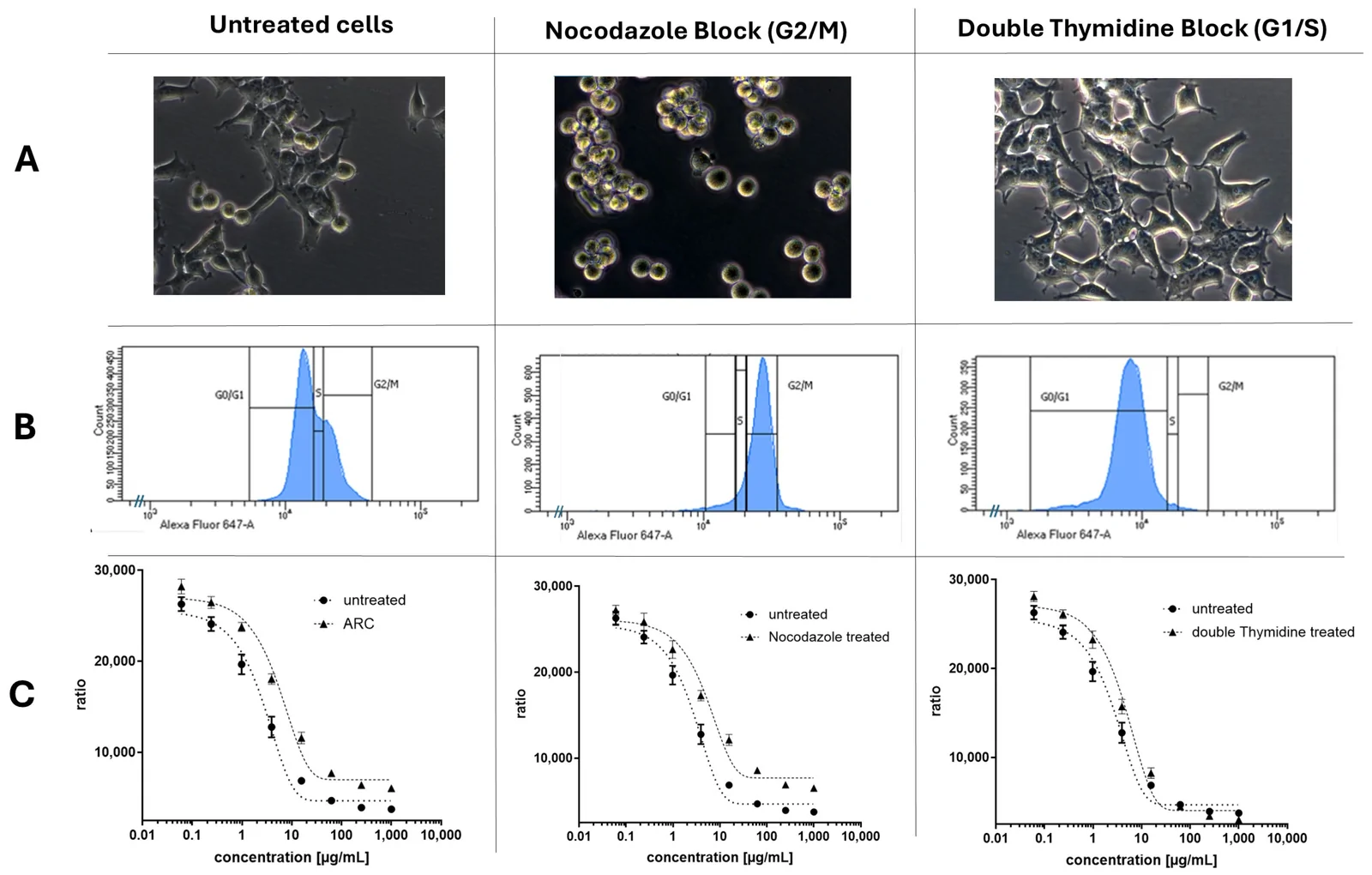
Analysis of HEK293 hGLP-1-R cells under different treatment conditions. Row (**A**): Representative images of untreated cells (left column), cells subjected to nocodazole block (middle column), and cells treated with a double thymidine block (right column). Row (**B**): Flow cytometry (FACS) profiles illustrating cell cycle distribution for each condition. Row (**C**): Dose–response curves in triplicate with SD after incubation of lixisenatide dilution series comparing untreated cells to assay-ready cells (first column) or to treated cells (2nd and 3rd columns) as analyzed with HTRF cAMP assay.

To enhance assay robustness through selection of lixisenatide-responsive cells, cell cycle synchronization was performed by arresting cells at defined cell cycle phases. Cultured cells were synchronized using nocodazole to block progression at the G2/M phase or double thymidine treatment to arrest cells at the G1/S phase. Dose–response analyses revealed no significant differences attributable to cell cycle stage (see Figure 4), indicating consistent GPCR-mediated signaling across all cell cycles.

Additionally, the general applicability of the HTRF cAMP assay was confirmed by successfully testing alternative GLP1 agonistic substances in the assay.

Statistically, an equivalence approach was established to assess parallelism, and a criterion of 0.8–1.25 for the assay control ensures meaningful potency determination.

In summary, the implementation of recent technologies—including assay-ready cells and streamlined assay protocols—combined with meaningful statistical criteria, contributed to reducing inherent assay variability and enhancing precision and accuracy.

### 2.2. HTRF cAMP Validation

Following updated ICH-Q2(R2) and USP<1033> guidance, a full assay validation includes repeatability assessment at five potency levels and intermediate precision considering the most critical assay parameters.

**Figure 5:**
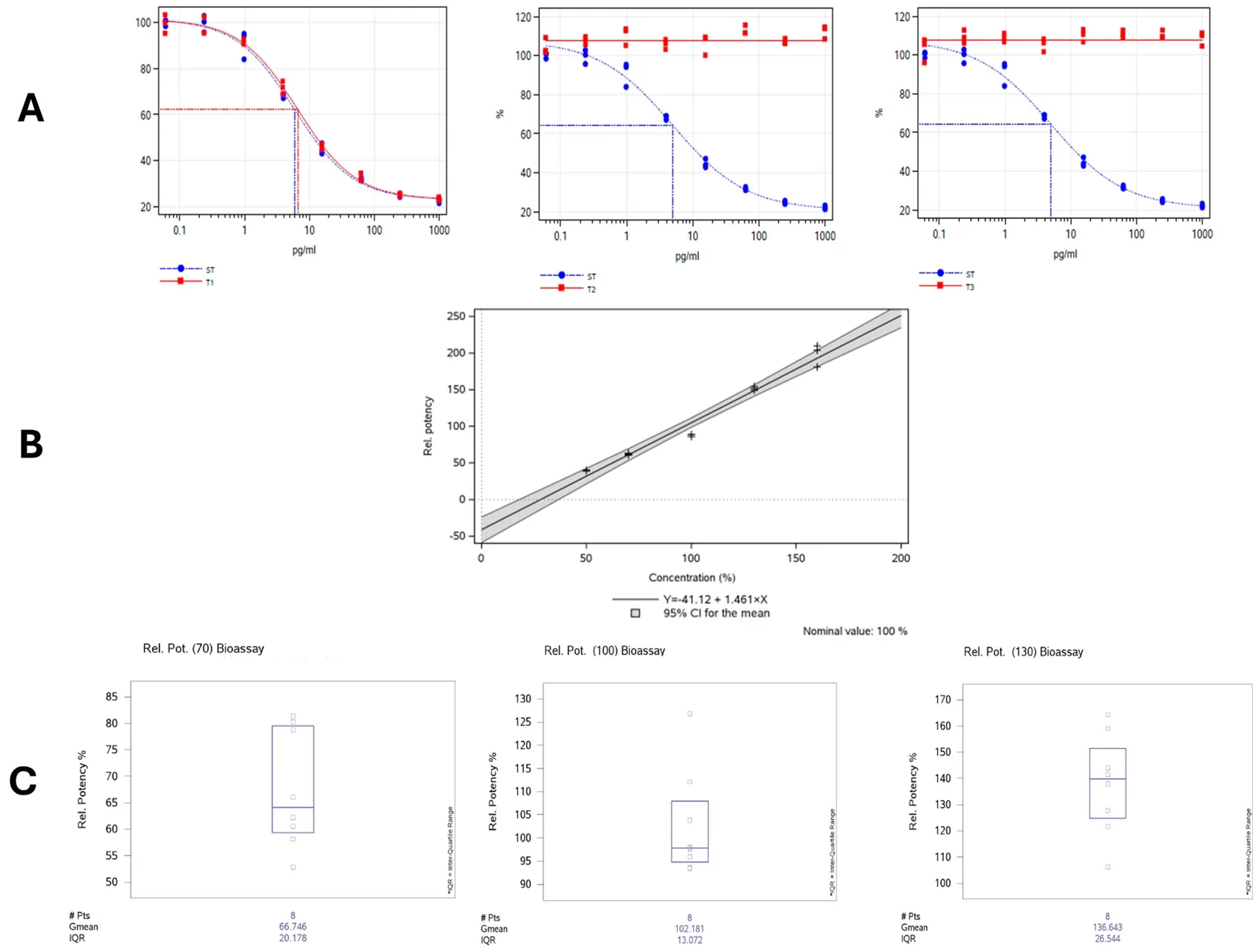
Lixisenatide cAMP HTRF assay validation. (**A**) Reference standard was tested against a 100%-potent T1 sample (left). No specific activity was observed for sample buffer T2 (middle), and the Des-[His(1)-Asp(9)]-lixisenatide T3 sample (right) demonstrated specificity for lixisenatide. (**B**) Linearity analysis using repeatability data demonstrated reliable correlation, with between 50 and 160% potency. (**C**) Intermediate precision determination at 70%, 100% and 130% potency. A total of eight determinations (Pts) derived from three assay plates, each in triplicate, were plotted against the relative potency (*y*-axis). The geomean and the interquartile range values are given as numbers and visually.

As outlined in Table 2 below, repeatability was determined by testing five different potency levels (50%, 70%, 100%, 130% and 160%), with three determinations per test sample performed by one analyst, using one cAMP HTRF test kit and assay-ready cells. One determination represents the mean of three assay plates, each in triplicate. Repeatability was determined to be 4% CV with a one-sided 95% confidence interval of 6%. Intermediate precision was determined by testing three key factors—analyst, different assay-ready cell banks, and different cAMP HTRF kits—at 70%, 100%, and 130% relative potency levels in a full factorial design with eight determinations in total. Intermediate precision and the 95% CI were calculated at each potency level using the internal software BioSt@t-Stars v2.7.2: 18% CV (34% at 95% CI) for PL70, 11% (21% 95% CI) for PL100 and 15% (29% 95% CI) for PL130 (see Table 1 and Figure 5C).

Five different dilution series of lixisenatide samples with 50%, 70%, 100%, 130% and 160% of the nominal concentration were used for the determination of dilutional linearity. The resulting relative potencies were plotted against the theoretical concentration in a linear regression using the software BioSt@t-Stars. The acceptance criterion for linearity (R^2^ ≥ 0.95) was met, as R^2^ was determined to be 0.9680 (see Table 2, Figure 5B).

Since no cellular response was observed using the N-terminally truncated version of lixisenatide [Des-[His(1)-Asp(9)] and sample buffer, while lixisenatide was detected to be active, the method is considered specific for lixisenatide (see Table 2, Figure 5A).

### 2.3. In Vivo Cross-Verification of cAMP Assay Data

To verify that cellular cAMP data correlate with in vivo effects, chemical lixisenatide variants were tested with the cAMP ELISA and an oral glucose tolerance test in a female db/db mouse test system. The chemical variants of lixisenatide result from amino acid modifications within lixisenatide: truncations (des variants), chemical alterations (including cyclic imidazole, isoaspartic acid, or N-acetylation), and duplications (di variants). These structural changes consequently modify the molecule’s biological activity.

The table below (Table 3) summarizes the results obtained with the in vitro cAMP ELISA assay and with the in vivo db/db mouse assay. Using both assays, lixisenatide and Exendin-4 were characterized as active. The byproducts show a heterogeneous biological activity.

**Figure 6:**
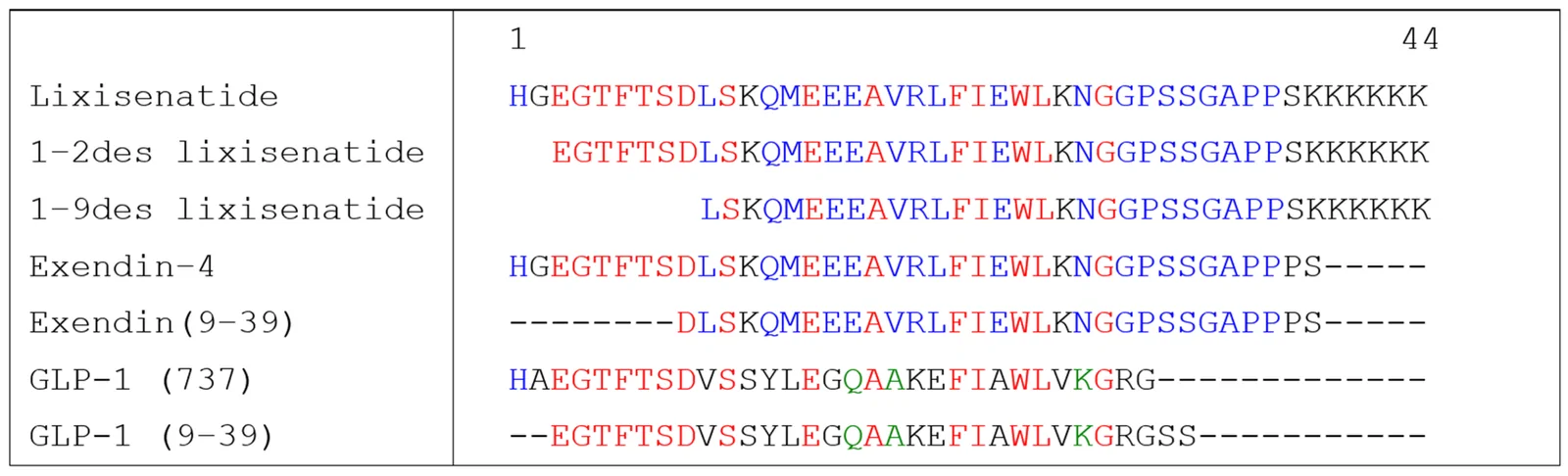
Amino acid sequence alignment (lixisenatide, GLP-1, GLP-1 analogous exendin and their truncated versions).

The first two N-terminal amino acids (see Figure 6) are essential for the agonistic activity of the GLP-1 agonistic substances. Therefore, the truncated versions GLP-1 (9–39) and des[His(1)-Gly(2)]-lixisenatide are expected to show reduced activity, whereas Exendin (9–39) and des[His(1)-Asp(9)]- lixisenatide are considered inactive in terms of GLP-1 receptor activation. N-terminally modified lixisenatide variants (cyclic-imide-Asp(9)- lixisenatide, Des-Gly(4)- lixisenatide, and Di-Leu(10)- lixisenatide) showed reduced or no activity, as did lixisenatide multimers (high-molecular-weight protein–lixisenatide = HMWP–lixisenatide), which represents non-covalently bound dimers via the N-terminal part of lixisenatide.

The correlation between the in vitro and the in vivo assay was assessed through categorical classification (active or non-active), as indicated by “Activity” in Table 3. In order to quantify the comparison of active results, a *t*-test based on log10-transformed data and the corresponding ratio of geometric means with 90% confidence interval calculation was performed. While Lixisenatide, cyclic-imide-Asn(28) and L-iso-Asp(28) revealed no significant differences (1.00 included in the confidence interval), di-Pro(36)-di-Pro (37), des-Ser(33) showed moderate differences and N-Acetyl-His (1), di-Pro(36) and di-Ala(35) significant to highly significant differences. Due to the very high method variability of the db/db mouse test, a clear qualitative correlation is not possible, which is confirmed by ratio calculation, where five substances met the bioequivalence criteria of 0.8–1.25 (as defined in USP<1090>). For lixisenatide as the reference compound, the complete confidence interval is included in the bioequivalence range.

Nevertheless, considering the high methodological variability, a correlation between in vitro and in vivo data based on the categorical classification was observed.

## 3. Discussion

Cell-based bioassays play a crucial role in the pharmaceutical industry, serving multiple purposes, including quality control release of active pharmaceutical ingredients and finished drug products, long-term stability assessment, evaluation of comparability between batches or manufacturing processes, and qualification of reference standards [20,21,22,36]. Among quality control methods, potency assays are unique in their capacity to measure biological activity, which is dependent on the primary structure as well as the integrity of complex three-dimensional molecular structures in solution [37]. Structure–function relationships, which are essential to product understanding, are primarily driven by bioassay studies on stressed samples [38,39].

Consequently, a significant emphasis must be placed on maximizing the precision of these assays to ensure the generation of meaningful and reliable data.

Lixisenatide was used as the representative GLP-1 agonistic substance.

All commercially available GLP-1 receptor agonists—including Exendin-4-derived compounds (lixisenatide and exenatide) and human GLP-1-derived compounds (liraglutide, semaglutide, dulaglutide, and albiglutide)—share the same binding site on the GLP-1 receptor [40] and display comparable receptor affinities in the low nanomolar range [41]. Based on these shared pharmacological properties, lixisenatide was selected as a representative compound for this drug class.

Notably, structural differences at the C-terminus of Exendin-4 may confer a marginally higher affinity for the GLP-1 receptor compared to native human GLP-1 [40,41]; however, whether these differences translate into distinct downstream signaling or altered receptor internalization remains to be elucidated. Comparative analysis of the downstream signaling effects induced by Exendin-4 and human GLP-1 demonstrated similar levels of cAMP activation, despite Exendin-4 exhibiting significantly higher receptor binding affinity [42].

The main distinguishing feature among GLP-1 receptor agonists is their differential susceptibility to DPP-4-mediated proteolytic degradation [43], which gives rise to the observed variations in in vivo half-life. However, these pharmacokinetic differences are not anticipated to affect the outcomes of the in vitro or in vivo assays utilized in the present study.

GLP-1 triggers multiple intracellular effects, including activation of signaling pathways via the GLP-1 receptor, Gs protein, and phosphodiesterase (PDE), thereby affecting the concentration of cAMP, protein kinase A (PKA) or MEK kinase, as well as intracellular Ca^2+^ release. The kinetics of the reaction cascade as well as the activities of adenylate cyclases (ACs) and PDEs lead to variable assay results when testing therapeutic compounds for their biological activity [44,45].

By using Lixisenatide as the representative GLP1 agonist, we found that all proposed in vitro assays demonstrated a dose-dependent effect of GLP1 agonistic activity when applied to the assays—independently of the cellular event (cAMP quantification, Ca^2+^ release, or β-Arrestin recruitment)—with varying levels of accuracy and precision.

The cAMP MSD assay exhibited higher variability, indicating that further optimization and development of this assay system is required to achieve acceptable performance. Specificity was successfully demonstrated across all assay platforms during the pre-validation phase, confirming the selective detection capabilities of each method. Linearity assessment revealed acceptable performance ranges, and precision evaluation showed comparable and acceptable performance across all in vitro test systems. Notably, cAMP ELISA was tested for intermediate precision with varying critical parameters. Accuracy determination revealed systematic biases characteristic of specific platforms: cAMP ELISA demonstrated a consistent negative bias, while both the PathHunter^®^ β-Arrestin assay and cAMP Glo™ exhibited positive biases. The PathHunter^®^ and cAMP Glo™/Ca^2+^ release systems showed increased variability at the lower and upper end of the tested working ranges. In contrast, cAMP HTRF generated data clustering around zero bias. Despite these platform-specific deviations, all test systems achieved acceptable accuracy within their respective operational parameters.

Importantly, we discovered that all assay set-ups measuring immediate cell responses matched well with set-ups measuring longer-term effects or, as shown later, fit well with in vivo data.

Early events read out might differ significantly to late read outs after receptor stimulation. A temporal bias within the GPCR signaling cascade has been reported [46]. The kinetics of early second messengers (such as Ca^2+^ flux within seconds or cAMP within minutes) differ from late biological responses (such as gene activation or cell survival after hours). It was demonstrated that a compound can act as a full activator in an “early readout” while showing minimal or no effect in a “late readout” (or vice versa).

Moreover, differential activation kinetics have been documented within GPCR signaling cascades [46]. Downstream readouts, including ERK1/2 phosphorylation, β-arrestin recruitment, cAMP generation, Gαi1 and GαoB protein activation, and dynamic mass redistribution (cellular impedance) demonstrated pathway-specific temporal kinetics and distinct maximal response amplitudes [47].

These signaling differences might lead to different robust cell-based potency assays. Therefore, various GLP-1R signaling cellular readouts were selected to examine whether differential responses could be observed upon receptor activation.

We discovered comparable performance across early and late signaling assays for GLP-1R activation. Furthermore, real-time measurements of Ca^2+^ release yielded results consistent with those obtained from endpoint cell-based assay (CBA) systems, without demonstrating a significant improvement in assay performance. All in vitro test systems were individually developed and optimized, which might explain these comparable potency data.

The cAMP HTRF assay set-up was chosen for further assay development, followed by an ICH Q2/USP<1033>-compliant validation. The experimental configuration was systematically optimized for improved precision and accuracy. Subsequently, a single-day assay format was developed utilizing assay-ready cells (ARCs) [48,49], and meaningful statistical criteria were established. The robustness of cellular signaling responses was confirmed through comprehensive cell cycle arrest investigations. Finally, extensive validation was conducted to establish and verify the cAMP HTRF method for routine application. The assay demonstrated accuracy within −3.5% to 6.0% CV across different potency levels, with repeatability consistently below 5%. Intermediate precision remained below 20%, whereas the 95% confidence interval reached up to 30%, which represents the typical cellular assay variability. These findings are consistent with FDA expectations [34] and literature reports, e.g., [32,33].

Successful cross-verification between the cAMP assay and the in vivo test system was demonstrated. Byproducts and truncated variants were evaluated using cAMP accumulation as an intracellular readout and verified in db/db mice to confirm their representativeness of the in vivo mode of action.

Human ex vivo and 3D platforms have been developed to investigate aspects of GLP-1 biology, for example, the secretion of human GLP-1 from intestinal tissues [50]. However, the blood-glucose-lowering effect of GLP1 still needs to be investigated with animal studies. In the present study, the oral glucose tolerance test (OGTT) in female db/db mice was selected to evaluate GLP-1 agonistic activity in vivo. Female db/db mice exhibit less severe glucose intolerance than males [51]. While this may reduce sensitivity for detecting glucose-lowering effects, it provides a model with preserved metabolic reserve that may better reflect early-stage type 2 diabetes.

These data nicely confirm public knowledge [35], which reports that the N-terminus is responsible for receptor activation. The loss of N-terminal amino acid residues was shown to result in a complete loss of functionality, as evidenced by the des1–2, des1–9, cyclic imidazole Asp9, and des Gly4 variants. N-terminal modifications, including acetylation of the first histidine residue, were found to impair GLP-1R binding, an effect detectable by the cellular assay but not by the in vivo test system, suggesting the superior sensitivity of the cellular assay. Furthermore, duplication of the leucine residue at position 10 led to a loss of GLP-1 agonistic activity in both test systems. In contrast, duplication of proline at positions 36 and 37 or alanine at position 35, or deletion of serine at position 33 had no significant impact on GLP-1 agonistic activity.

Nevertheless, some limitations must be considered. The restricted number of GLP-1 receptor agonists evaluated—Lixisenatide only—might constrain the interchangeability of these findings to the other GLP-1-based therapeutics. The experimental design focused exclusively on immediate in vitro and in vivo responses, precluding assessment of long-term pharmacodynamic effects that may be clinically relevant. This emphasis on acute signaling events may not fully recapitulate the sustained metabolic benefits observed with chronic GLP-1 receptor agonist therapy. Although cAMP accumulation demonstrated robust performance as a functional readout in the present study, this endpoint may lose its predictive validity if structurally divergent GLP-1 receptor agonists preferentially activate alternative downstream signaling cascades or exhibit biased agonism.

Therefore, confirmation with structurally diverse compounds (e.g., dual agonist molecules) and verification using complementary functional assays are recommended to ensure the relevance of this assay system prior to its implementation as a quality control test for other GLP-1 therapeutic compounds.

## 4. Materials and Methods

**Lixisenatide dilution series.** The assays were developed with eight to twelve distinct lixisenatide concentrations per dilution curve. The standard and sample were prediluted in low-binding tubes in triplicate, followed by a serial dilution in a 96-well plate. The final dilutions from one dilution plate were transferred to the cell plates.

For assay comparison and pre-validation, relative potency was calculated based on a single 96-well cell plate containing sample triplicates. In contrast, final assay validation results for the HTRF cAMP assay were derived from the geometric mean of three independent relative potency measurements, each obtained from a separate cell plate containing sample triplicates.

**Human Embryonic Kidney 293 cells (HEK293- hGLP-1-R):** Embryonic Kidney 293 cells expressing the human GLP-1 receptor (GeneBank accession number: NM_002062) were cultivated with 90% DMEM with GlutaMAX and 4.5 g glucose (Gibco, #1965 by Thermo Fisher Scientific, Waltham, MA, USA), 10% fetal bovine serum (Invitrogen, #10082 by Thermo Fisher Scientific, Waltham, MA, USA), 1× MEM non-essential amino acids (Invitrogen, #11140035) and 1 µg/mL Puromycin (Sigma Aldrich, #P9620 by MilliporeSigma, Burlington, MA, USA) at +37 °C in a humidified 5% CO_2_ atmosphere.

**cAMP MSD Assay:** The commercially available cAMP assay from MSD (Meso Scale Discovery, Rockville, MD, USA) detects the binding of GLP-1 or a GLP-1 agonist to the GPCR via the intracellular signaling molecule cAMP. The assay follows the principle of a competitive immunoassay based on the displacement of TAG-labeled cAMP.

All buffers as well as the anti-cAMP pre-coated 96-well plates were delivered with the cAMP MSD Kit (Meso Scale Diagnostics (MSD), cat. #K150FDD-2).

In the first step, 20,000 HEK293-hGLP-1-R cells were prepared in a volume of 20 µL per well. The cells were resuspended in cell buffer (assay buffer contained in the kit with 1 mM IBMX (Calbiochem, #410957)). The corresponding dilutions of lixisenatide in the range of 20,000–0.3 pg/mL were prepared, and 10 µL per well was added to the plate together with the cells. To increase the volume, an additional 20 µL of assay buffer was added per well and incubated for 30 min with gentle shaking. Subsequently, 20 µL of Sulfo-TAG™ solution (TAG-cAMP contained in the kit dissolved in lysis buffer) was added per well and incubated for another 2 h at room temperature with shaking. Immediately before measurement using the MSD MESO QuickPlex SQ 120, 100 µL of 1.5× Read Buffer (included in the kit) was added per well.

**cAMP Glo™ Bioassay:** The commercially available cAMP-Glo™ Assay from Promega (Madison, WI, USA) detects the binding of GLP-1 or a GLP-1 agonist to the GPCR based on the formation of cAMP. Detection is possible through a bioluminescence signal.

For the implementation of the cAMP-Glo™ Assay, lixisenatide in the range of 1250–0.3617 pg/mL and cells were diluted or resuspended in induction buffer (D-PBS (Gibco, #4190-094) with 500 µM IBMX (Calbiochem, #410957) and 100 µM Ro 20-1724 (Sigma-Aldrich, #B8279).

A total of 15,000 HEK293-hGLP-1-R cells were plated per well with a volume of 30 µL. For treatment of the cells, 10 µL of lixisenatide was added per well and incubated for 30 min. Cell lysis was performed by adding 20 µL of lysis buffer (provided with Promega cAMP Glo™ Assay Kit #V150) for 30 min with gentle shaking. For detection, the cAMP-Glo Detection Solution (cAMP Glo™ Assay Kit) was prepared according to the manufacturer’s instructions, and 41 µL per well was added to the plate. After briefly shaking the plate for about 1 min, the plate was incubated for 20 min at room temperature. After subsequent addition of 80 µL Kinase-Glo Reagent (cAMP Glo™ Assay Kit) per well and brief shaking of the plate, another incubation of 10 min at room temperature was performed with a light-impermeable lid. The luminescence signal was then analyzed on the microtiter plate reader with an integration time of 1000 ms.

**cAMP Homogeneous Time-Resolved Fluorescence Assay:** The commercially available cAMP Homogeneous Time-Resolved Fluorescence Assay (cAMP GS dynamic kit (#62AM4PEC, Cisbio Bioassays, Codolet, France)) is also based on the activation of adenylyl cyclase through GPCR activation after the binding of GLP-1 or a GLP-1 agonist.

HEK293-hGLP-1-R cells were resuspended in cell buffer (20 mM HEPES (Gibco, #5630)), and 5,000 cells were plated with a volume of 25 µL per well in white 96-well half-area plates (#693, Corning Incorporated (Costar), Arlington, VA, USA). Lixisenatide was diluted in stimulation buffer (1% BSA (Sigma, #A9647) and 1 mM IMBX (Calbiochem, #410957) in cell buffer) to the desired concentration in LoBind tubes (#22431081, Eppendorf, Hamburg, Germany).

For treatment of the cells, 25 µL of lixisenatide solution in the range of 250–0.02 pg/mL final concentration per well was added and incubated for 30 min at room temperature. Cell lysis and detection was performed by adding 25 µL of 1× cAMP-d2 reagent solution followed by 25 µL of 1× anti-cAMP-cryptate solution (delivered with GS dynamic kit). The 96-well plate was sealed and incubated for 1 h at RT without shaking. The read-out was performed on a 96-well-plate HTRF-compatible fluorescence reader.

**PathHunter^®^ β-Arrestin Assay:** The commercially available PathHunter^®^ β-Arrestin Assay from DiscoverX (Eurofins DiscoverX, Fremont, CA, USA ) detects GPCR receptor activation by GLP-1 or a GLP-1 agonist through the interaction with β-arrestin and the resulting β-galactosidase enzyme fragment complementation.

For the PathHunter^®^ β-Arrestin Assay, 10,000 β-Arrestin-CHO-K1-GLP-1-R cells (delivered as ready-to-use cells by DiscoverX (t#3-0300C2)) per well were plated in 100 µL assay medium (90% F-12 Nut Mix (1×) with GlutaMAX (Gibco, #31765-027), 10% FBS (Invitrogen, #10082) and 1× Pen/Strep (Sigma-Aldrich, #P0781)) in a 96-well cell culture plate and incubated for 24 h at 37 °C and 5% CO_2_. For the treatment of cells with lixisenatide, serial dilutions of lixisenatide in D-PBS + 0.5% BSA in the range of 1.818–0.00014 µg/mL were added to the cells in a volume of 10 µL per well and incubated for 90 min at 37 °C and 5% CO_2_. A detection kit (DiscoverX, #3-0001) was used for cell lysis and detection in one step. Here, 55 µL working detection solution per well was added to the treated cells. After an incubation period of one hour at room temperature with a light-impermeable lid, luminescence measurement was performed on the microtiter plate reader with an integration time of 1000 ms.

**Analysis of GLP-1 cell-based assay data:** For the statistical analysis of the dose–response relationship, the signals were plotted against the lixisenatide concentration. The semi-logarithmic plotting of the results yielded a sigmoidal curve. The dilution curves of a lixisenatide reference standard and a lixisenatide test sample were used to perform a 4-parameter logistic (4-PL) regression analysis and to calculate the EC_50_ or IC_50_ values with the half-maximal effective concentration, which represents the potency of lixisenatide. The relative potencies were calculated based on dividing the E/IC_50_ value of a reference standard by the E/IC_50_ value of the sample, which was then multiplied by 100%.

$$\%\;relative\;potency=100*\left(\frac{E/{IC}_{50}\;standard}{E/{IC}_{50}\;sample}\right)$$

For determining parallelism and system precision, the CV[%] of E/IC_50_ was determined to be ≤20%; a *p*-value/F-test of ≥0.05 proves that the two curves are not different, and the coefficient of determination R^2^ must be ≥0.98. In case of a *p*-value < 0.05, the coefficient of determination (R^2^) must be ≥0.99.

**cAMP ELISA using HEK293-hGLP-1-R (#1629):** The #1629 cell line is a derivative of HEK293 cells modified with the human G-protein Gα16 gene and the human GLP-1-R gene. The GLP-1 receptor was activated, and subsequently cAMP production was induced in a dose-dependent manner and measured using a commercial cyclic AMP assay ParameterTM ELISA Kit (#KGE002B, R&D Systems, Minneapolis, MN, USA). This lixisenatide assay system was deployed at Cerbaresearch, Laval, Canada.

PSCGa16GLP-1R #1629 cells were resuspended in culture medium (90% D-MEM with GlutaMAX™ (Invitrogen, #10566024), 10% FCS (#A15-251, Lonza (formerly PAA), Pasching, Upper Austria, Austria), 1× MEM non-essential amino acids (Invitrogen, #1140-050), 1 µg/mL pyromycin (Sigma-Aldrich, #P7255) and 250 µg/mL Zeocin (Invitrogen, #R250-01)), and 50,000 cells/well were plated in two 96-well plates (Corning, #365). The cell culture medium was aspirated from each well, and 200 µL of freshly prepared stimulation buffer (0.1% BSA (PAA, #K41-001) and 1 mM of IBMX (Sigma-Aldrich, #I5879) in 1× HBSS (Invitrogen, #4185)) was added to each well and incubated at 37 °C in the 5% CO_2_ incubator for 30 min. Lixisenatide standard was diluted in stimulation buffer in the range of 350–7.8 pg/mL and the testing solutions to 100/75 and 50 pg/mL in lobind tubes (Eppendorf, #CA80077-232). The stimulation buffer was aspirated from each well, and 100 µL of the appropriate lixisenatide solution or control was added to each well; the plates were incubated at 37 °C in the 5% CO_2_ incubator for 30 min. After incubation, the supernatant was aspirated from each well and the plate was washed 3 times by adding 200 µL of cold PBS buffer. After aspiration and adding 200 µL 1× cell lysis buffer 5 (from cAMP ELISA kit) to each well, the plate was frozen at ≤−70 °C for at least 45 min. After thawing, the freeze/thaw cycle was repeated 3 times. Finally, cells were thawed with gentle mixing for at least 45 min and centrifuged at 600× *g* for 10 min at 2–8 °C to remove cellular debris. Then, 150–175 mL of supernatant was collected in microtubes and the cyclic AMP ELISA was performed according to the manufacturer’s instructions.

The net OD values (OD450-550) were averaged for all replicates, and the %bound in correlation with the negative control was calculated. A four-parameter logistic equation (4PL) was used for the lixisenatide standard, and the lixisenatide samples were quantified by back-calculation using the 4PL curve of the standard. The %CV of the replicates and the relative bias of control samples (low QC, mid QC and high QC) were used to prove system suitability.

**Ca2+ release test (FDSS µcell by Hamamatsu Photonics, Hamamatsu, Shizuoka, Japan):** HEK293-hGLP-1-R cells and the FLIPR Calcium5 -Assay-Kit (#R8186) from Molecular Devices (San Jose, CA, USA) were used. The kit utilizes different dyes: while one dye acts as a quencher to reduce the background signal, a second dye, a calcium-sensitive fluorescence dye, is also used, which can be measured only inside the cells. By stimulating GLP-1-R via GLP-1-agonist, the calcium ion channels open and calcium gets into the cell. This calcium accumulation can be measured in real time using the Hamamatsu FDSS µcell system.

Lixisenatide dilution series were prepared with assay buffer (20 mM HEPES (Gibco, #15630056) in 1× HBSS (ThermoFisher Scientific, #14185052)) in 384 PP plates (#781281, Greiner Bio-One, Kremsmünster, Austria) ranging from 3.33 104 to 16 pg/mL final concentration in 12 dilution points.

HEK293-hGLP-1-R cells were resuspended in cell buffer (20 mM HEPES (Gibco, #5630)), and 15,000 cells were plated in a volume of 25 µL per well in polystyrole 384-well plates (Greiner, #781091) and incubated for 5 min at 37 °C, 5% CO_2_, in a humid environment. Then, 25 µL of diluted reagent A from the FLIPR^®^ Calcium 5 Assay Kit reconstituted in assay buffer was added to each well and incubated for at least 30 min at 37 °C, 5% CO_2_, in a humid environment. The addition of the lixisenatide dilution series to the cellular/substrate plate was carried out by the Hamamatsu FDSS µcell device.

For the calculations, the blank value was subtracted, and the highest peak value was used for further analysis. The height of calcium release was correlated with the lixisenatide concentration to obtain a sigmoidal dose–response curve. A 4-parameter logistic (4-PL) regression analysis to calculate the EC_50_ value was performed. The relative potencies were calculated by comparing the EC_50_ value of a reference standard with the EC_50_ value of a sample.

**Cell cycle arrest:** A total of 1 × 105 HEK293- hGLP-1-R cells were seeded in a T75 flask and incubated for 72 h +37 °C in a humidified 5% CO_2_ atmosphere. After adding thymidine solution (Sigma-Aldrich, #T9250-1G) at a final concentration of 2 mM, the cells were further incubated for 18 h at +37 °C in a humidified 5% CO_2_ atmosphere. After washing with PBS, the cells were again incubated for 9 h at +37 °C in a humidified 5% CO_2_ atmosphere, followed by incubation for an additional 18 h with thymidine solution at a final concentration of 2 mM to fix the cells at the G1/S boundary phase. After washing with PBS by centrifugation at 500× *g*, the cells were frozen until further analysis [52].

A total of 3 × 105 HEK293- hGLP-1-R cells were seeded in a T75 flask and incubated for 72 h at +37 °C in a humidified 5% CO_2_ atmosphere. After adding nocodazole solution (Sigma-Aldrich, #SML1665-1ML) at a final concentration of 400 ng/mL, the cells were incubated for 16 h at +37 °C in a humidified 5% CO_2_ atmosphere. After centrifugation at 500× *g*, the cells were frozen until further analysis (see [53,54]).

**Fluorescence-Activated Cell Sorting (FACS)—analysis:** For cell cycle analyses, Draq5™ (#424101, BioStatus Limited, Llanelli, UK ) as DNA dye and Alexa Fluor™ 488 as dye coupled on a GLP1 monoclonal antibody (R&D Systems,. #FAB2814G) and 7-Amino-Actinomycin D (7-AAD) (#51-68981E, BD Biosciences, Erembodegem, Belgium) were chosen. Cryopreserved cells were thawed rapidly in 37 °C water and immediately transferred to 15 mL conical tubes containing 10 mL of FACS washing buffer (10% FBS in D-PBS). Then, 1 × 10^5^ cells were transferred into individual FACS tubes and the volume was adjusted to approximately 2 mL with FACS staining buffer (2% FBS in D-PBS). After centrifugation at 200× *g* for 5 min, cell pellets were resuspended in 100 µL of FACS staining buffer containing either DRAQ5™ nuclear stain, isotype control antibody, or primary GLP-1R antibody. Tubes containing GLP-1R antibody or isotype control were incubated for 20 min at room temperature protected from light, followed by 2× washing with 2 mL FACS staining buffer (centrifugation at 200× *g* for 5 min). Tubes containing DRAQ5™ were incubated for 12 min at room temperature, protected from light, without subsequent washing. Unstained control cells received no incubation period. All samples were 2 × washed, and cell pellets were resuspended in 300 µL FACS staining buffer and analyzed immediately using a FACS Canto™ II (BD Biosciences operated with FACSDiva v8.0.1).

**Biostatistical calculations:** For the determination of accuracy, the relative bias (RB) and its associated 90% confidence interval (CI RB;LL/UL) was calculated using the equations below.

Relative bias (RB) calculation:

$$RB\;[\%]=100*\left(\frac{GM}{TC}-1\right)\%$$

GM = geometric mean and TC = theoretical concentration.

Calculation of 90% confidence interval (CI_RB;UL/LL_) of the relative bias:

$${CI}_{RB,LL/UL}[\%]=100*\left(\frac{mean\pm t_{df}*\frac{SD}{\sqrt{n}}}{TC}-1\right)\%$$

LL/UL = lower limit and upper limit; 

mean = mean of measurements;

n = number of measurements;

tdf = statistical constant with n-1 degrees of freedom;

SD = standard deviation;

TC = theoretical concentration.

Repeatability or intermediate precision (Rep/IP) and its associated 95% confidence interval (CI_95;Rep/IP_) was calculated using the equations below.

Repeatability/Intermediate precision calculation:

$$Rep/IP\;\left[\%\right]=\left(10^{\frac{CV\;\left[\%\right]}{100}*mean}-1\right)*100$$

CV[%] = coefficient of variation.

Calculation of 95% confidence interval (CI_95;Rep/IP_):

$${CI}_{95;IP}\left[\%\right]=\left(10^{\left(\frac{{CI}_{95,CV\left[\%\right]}}{100}*mean\right)}-1\right)*100$$

**Oral glucose tolerance in female db/db mice:** Female diabetic db/db (BKS.Cg-m +/+ Leprdb/J) mice (7 weeks of age) were obtained from Charles River Laboratories, Sulzfeld, Germany and maintained under standardized animal house conditions on standard rodent diet until study start.

The animal facilities were AAALAC-certified, and the animal studies were conducted according to the German Protection of Animals Act and under control of the Regional Counsil of the State of Hesse.

In separate experiments, at 9–13 weeks of age, when the animals were overtly diabetic, the effect of single-dose treatment with either lixisenatide or its isolated synthesis impurities or degradation products was evaluated in the oral glucose tolerance test. The animals were fasted for 18 h prior to injection of the test compounds and had free access to tap water.

Before the start of the study, the animals were randomized to groups by the investigator. To achieve an acceptable level of statistical confidence, a minimum of n = 5–10 replicates was included in the analysis.

The diabetic db/db mice received a single subcutaneous injection of a placebo (400 µL/kg SC), lixisenatide (10 µg/kg SC or 2 nmol/kg SC), or equal doses of the respective synthesis impurities or the degradation product 30 min prior to an oral gavage of 2 g/kg glucose. All drugs and controls were administered subcutaneously at a volume of 400 µL/kg.

Blood glucose was determined from tail-tip blood taken before the first injection and subsequently up to 3 h after the oral glucose gavage (total 3.5 h). Blood glucose was determined enzymatically (Gluco-quant^®^ Glucose/HK kit on Roche/Hitachi 912, Roche Diagnostics, Mannheim, Germany).

To minimize bias, study responsibilities were distributed across independent personnel. Study planning and reporting were conducted by the Study Manager, experimental procedures were performed by a dedicated laboratory technician, and final data evaluation and statistical analysis were carried out by an independent statistician.

**Statistical analysis:** The total area under the curve (AUC) for each animal (AUC 30–210 min) was determined according to blood glucose levels for a time period of 30 min to 210 min using the trapezoid method.

Results are expressed as means ± SEMs for each treatment group. The 95% confidence intervals (CIs) of mean AUC levels are given, and significant differences versus control or lixisenatide group were assessed by comparing confidence intervals. Drug effects are expressed as the difference from the mean AUC of the control group and are given as a percent of lixisenatide gain:

$$\%\;Lixisenatide\;gain=100*\left(\frac{sample-control}{lixisenatide-control}\right)$$

Percent of Lixisenatide gains between 0% and 100% characterize gains between control and lixisenatide. Percent of lixisenatide gains beyond 100% characterize gains exceeding lixisenatide, and percent of lixisenatide gains below 0% characterize gains undercutting control, referred to as excess Lixisenatide gain.

## 5. Conclusions

The need to improve sensitivity, reliability, and representativity for the mode of action of a pharmaceutical drug has driven advancements in biological assays, exemplified by the development of cell-based bioassays to quantify the bioactivity of therapeutic GLP-1 agonist compounds described in this case study. All assay types targeting different cellular mechanisms were representative of GLP-1 agonistic compounds with different precisions and accuracy results. Ca^2+^ real-time measurement confirmed endpoint measurements of either cAMP accumulation or β-arrestin recruitment. These data were cross-verified with in vivo data, confirming the representatives of the in vitro assays.

This research helps to better understand how to reliably test these important medications. By improving our quality control methods, we can ensure that patients receive high-quality, effective treatments for a range of health conditions.

## Figures and Tables

**Table 1:** Assay pre-validation.

Validation Parameter		cAMP ELISA	cAMP HTRF	PathHunter^®^ β-Arrestin	cAMP Glo™	Ca^2+^ Release/FDSS µ Cell
Specificity	Analyte measurable in the presence of expected components, like impurities, degradants, and matrix	+	+	+	+	+
Linearity	Results are directly proportional to the concentration (amount) of analyte in the sample	−2.1 to −4.4% ^a^	R^2^ = 0.95	R^2^ = 0.87	R^2^ = 0.96	R^2^ = 0.96
Accuracy	Closeness between the measured value and the true (=expected) value or an accepted reference value	−22.8% to −0.1% ^b^	−14.5% to 4.5%	11.4% to 7.0%	−28.0% to 3.4%	−15.4 to 18.7%
Precision	Closeness between a series of measurements obtained from multiple measurements of the same sample	8.7 to 20.4% ^b^	8.8% ^c^	7.0% ^d^	7.7% ^d^	11.7% ^d^

^a^: linearity was demonstrated by bias determination of 2 concentrations in the dose–response curve. ^b^: accuracy and precision were determined at 5 concentration levels with DS and DP. ^c^: repeatability at nominal concentration with n = 8. ^d^: repeatability at nominal concentration with n = 6.

**Table 2:** cAMP HTRF assay validation.

Validation Parameter		Results
Specificity	Lixisenatide is replaced by Des-[His(1)-Asp(9)]-lixisenatide and buffer interference	No unspecific detection
Linearity	Linear regression determined to theoretical PL at 50-70-100-130-160 with R^2^ and y intercept	R^2^ = 0.97
Accuracy	Bias at PL 50-70-100-130-160 within +−20%CV *	−3.5% to 6.0%
Precision	Repeatability at 5PL with n = 3 within +−20%CV * Intermediate precision with a full factorial design considering the most critical parameters at PL 70-100-130 (analyst, ARC vs. cultured cells, incubation time) within +−30%CV *	4% PL70 18% PL100 11% PL130 15%

* Generic criteria have been applied based on published case studies [32,33] or authority guidance [34].

**Table 3:** In vivo assay cross-verification.

Substance Tested	cAMP ELISA			Oral Glucose Tolerance Test in db/db Mice		
	Activity	% Activity	RSD% ^a^	Activity	% Activity	SEM% ^b^
Placebo	−	<LOQ ^c^		−	0	13
Lixisenatide	+	93.9	6.6	+	100	7
des-[His(1)-Gly(2)]-lixisenatide	−	<LOQ ^c^		(−)	33	11
des-[His(1)-Asp(9)]-lixisenatide	−	<LOQ ^c^		-	−14	18
cyclic-imide-Asn(28)-lixisenatide	+	83.6	7.0	+	90	18
cyclic-imide-Asp(9)-Lixisenatide	−	<LOQ ^c^		(−)	39	13
L-iso-Asp(28)-lixisenatide	+	105.3	9.2	+	91	23
N-Acetyl-His(1)-lixisenatide	(−)	38.1	9.2	+	91	9
des-Gly(4)-ilxisenatide	−	<LOQ ^c^		−	3	14
di-Pro(36)-di-Pro(37)-Lixisenatide	+	110.8	7.4	+	91	6
di-Leu(10)-lixisenatide	−	<LOQ ^c^		−	14	21
des-Ser(33)-lixisenatide	+	103.5	9.7	+	72	18
di-Pro(36)-lixisenatide	+	123.4	3.7	+	139	11
di-Ala(35)-lixisenatide	+	99.2	5.4	+	105	13
HMWP-lixisenatide	−	<LOQ ^c^		(−)	39	20
Exendin-4	+	153.1	7.6	+ ^d^		
Exendin (9–39)	−	<LOQ ^c^		−	−17	12

Considering the high analytical variability, results above 60% activity were grouped as active substances (‘+’), those between 60 and 40% activity as partially active (‘(+)’), those between 40 and 20% as weakly active (‘(−)’), and those below 10% as negative (‘−’); ^a^: RSD: relative standard deviation in percent; ^b^: SEM: standard error of the mean; ^c^: <LOQ, activity was below the limit of quantification, which is <15.6 pg/mL; ^d^: according to the literature [35].

## Data Availability

The original contributions presented in this study are included in the article material. Further inquiries can be directed to the corresponding author.

## Abbreviations

The following abbreviations are used in this manuscript:

cAMP	Cyclic adenosine monophosphate
CV	Coefficient of variation
db/db mice	Diabetic mice
DPP-4	Dipeptidyl peptidase-4
ELISA	Enzyme-linked immunosorbent assay
FDA	U.S. Food and Drug Administration
GLP-1	Glucagon-Like Peptide-1
GPCR	G-protein coupled receptor
GTP/GDP	Guanosine triphosphate/guanosine diphosphate
HMWP	High-molecular-weight protein
HTRF	Homogeneous Time-Resolved Fluorescence
ICH	International Council for Harmonisation
IP_3_	Inositol 1,4,5-trisphosphate
LOQ	Limit of quantification
MEK	Mitogen-Activated Protein Kinase
MoA	Mechanism of action
MSD	Meso Scale Discovery
PKA	Protein Kinase A
PIP_2_	Phosphatidylinositol 4,5-bisphosphate
QC	Quality control
RSD	Relative standard deviation
SD	Standard deviation
SEM	Standard error of the mean
T2DM	Type 2 diabetes mellitus
USP	United States Pharmacopeia
